# Cognitive decline and poor social relationship in older adults during COVID-19 pandemic: can information and communications technology (ICT) use helps?

**DOI:** 10.1186/s12877-022-03061-z

**Published:** 2022-04-28

**Authors:** Yaya Li, Kayo Godai, Michiko Kido, Susumu Komori, Ryoichi Shima, Kei Kamide, Mai Kabayama

**Affiliations:** 1 Department of Health Sciences, Graduate School of Medicine, Osaka University, Osaka, 565-0871 Japan; 2 Health and Welfare Center, Toyono Town, Osaka, 563-0103 Japan; 3 Strategic Global Partnership & the X(Cross)-Innovation Initiative, Graduate School of Medicine / Faculty of Medicine, Osaka University, Osaka, 565-0871 Japan

**Keywords:** ICT use, Loneliness, Social isolation, Cognitive decline, Older adults, Japan

## Abstract

**Background:**

To answer whether older adults' cognitive function benefits from ICT use, we (1) examined the relationship between ICT use and cognitive decline during the COVID-19 pandemic and (2) explored the potential role of ICT use in mitigating the relationship between loneliness, social isolation, and cognitive decline among community-dwelling older adults.

**Methods:**

From February to March 2021, a mail survey was distributed to 1,400 older adults aged 70–89 years old. Responded participants were 1,003 (71.6% response rate). Subjective cognitive decline (SCD) was the independent variable. ICT use was assessed based on ICT use history and current ICT use activities. Loneliness was based on the Japanese version of the Three-Item Loneliness Scale. Social isolation was a total score of six items. Covariate-adjusted logistic regressions were performed and stratified by age groups (70–79 and ≥ 80 years).

**Results:**

During the COVID-19 epidemic, the proportion of people aged ≥ 80 years who reported cognitive decline was twice that of 70s. Non-ICT use was independently associated with a higher risk of cognitive decline in participants aged ≥ 80 years. Furthermore, the significant associations between cognitive decline and interaction items (non-ICT use by loneliness or social isolation) were observed in the ≥ 80 age group. No association was found in the 70–79 age group.

**Conclusions:**

Non-ICT users with high loneliness or social isolation scores were more likely to experience cognitive decline for adults age ≥ 80 years. For older adults who were vulnerable to poor social relationships, ICT use is potentially an efficient intervention. Further longitudinal investigations are needed.

**Supplementary Information:**

The online version contains supplementary material available at 10.1186/s12877-022-03061-z.

## Background

Effective interventions for cognitive health during later life are critical considering the age-related cognitive changes [1]. Poor social relationships, which are commonly assessed by subjective loneliness or objective social isolation, have been suggested to be modifiable risk factors for adverse cognitive outcomes [2–9]. Older adults are especially vulnerable to poor social relationships [10, 11]. The COVID-19 pandemic future increased the risk of feeling longlines or being social isolated due to life changes including social distancing, less social participation, and limited social communication [12, 13], which in turn increases the risk of adverse cognitive outcomes. It raises a question that how to keep protecting cognitive health from loneliness and social isolation for older adults when social life is limited.

Recent newest researches started to notice the potential positive effects of information and communication technology (ICT) use on memory [14], cognitive function [15, 16] and cognitive decline [17, 18]. The use of ICT is also expected to be an efficient intervention tool against loneliness [19, 20]. In the information age, ICT is becoming increasingly widespread. Social distancing during the current COVID-19 pandemic further enriched the use of ICT and highlighted its importance. The expanded ICT use has also increased the risk of pushing older adults into a vulnerable position because of the digital divide [21, 22]. Clarifying the ICT use-cognition relationship among older adults is imperative to health equity and public policies for with/after COVID-19 era. Research is still growing, however, there is few evidence of the relationship between ICT use and cognitive function in an Eastern context [18], especially in super-aging Japan so far. Meanwhile, the interaction effect between ICT use and poor social relationship on cognitive health is less explored. This study explored the possible mitigate role of ICT use on the association between poor social relationships and cognitive health.

There are some theoretical mechanisms from the existing literature that potentially support the hypothesis. One is the cognitive reserve theory, which implies the presence of cognitive reserve-based individual differences in the efficiency of protection against age-related brain pathology [23]. Larger cognitive reserve can provide greater resistance to cognitive risks and better performance on cognitive tasks [24–26]. Cognitive reserve is a modifiable factor that could be diminished by risk factors such as loneliness or social isolation, but it could be stimulated by ICT use or ICT use-related factors like social interactions, intellectual activities, and leisure activities simultaneously [14, 17, 23, 27, 28]. The psychological pathway is another theoretical pathway linking poor social relationship and ICT use to cognitive health [8, 29, 30]. Loneliness and social isolation could lead to lower capacity for emotional self-regulation and risks for negative outcomes like depression, stress, feeling unsafe, low self-esteem, and low self-efficacy [31, 32], which in turn negatively affect health through risky behaviours or high cortisol levels [29, 31, 33]. On the other hand, ICT use seems to contribute to better sleep [15], lower depression [15], and a sense of empowerment [30] among older adults.

Traditional interventions to prevent cognitive decline in older adults, in particular the social interactions, are facing challenges in the current COVID-19 pandemic. Some studies started to treat ICT use as a useful intervention to maintain cognitive function [34]. Examining the possibility of linking ICT use to cognitive function among older adults may help deepen the understanding of disparities in health status due to the digital divide, potentially leading to new effective interventions when facing barriers to social interactions. The proportion of the population with dementia in Japan is about twice the world average [35]. However, evidence supporting an association between ICT and cognitive function in Japan is scarce. Therefore, during the third wave of the pandemic, we conducted a survey among older Japanese to examine relationships between ICT use and cognitive decline. Given the similar theoretical mechanisms relating social relationships and ICT use to cognitive function, we further assume that ICT use plays a mitigating role on the relationships between loneliness, social isolation, and cognition decline.

## Methods

### Data and participants

We collected self-reported questionnaire data in T-Town, a rural area in western Japan. Older adults aged ≥ 70 years were selected randomly from the residential registration system using stratified random sampling. We collaborated with the local government and sent 1,400 (about 20% of the whole population of 70–89 years old) anonymous self-administered questionnaires with informed consent forms by mail from February to March 2021 (during the later phase of third wave and under the emergency state in Japan). We explained in the questionnaire that this study is not compulsory and data will be analyzed without identifying individuals. In total, 1003 participants completed questionnaires (71.6% response rate).

### Ethics

The Osaka University Clinical Research Review Committee approved this study.

### Assessment of variables

#### Subjective cognitive decline (SCD)

SCD is a self-reported experience of worsening. It is viewed as a predictor of future cognitive decline [36–38] and an early sign of dementia [39, 40]. It may indicate an initial change in cognitive function that cannot be detected by objective tests [36]. Participants were asked a single question that “Do you feel rapid cognitive decline?” and shown a four-point scale (Strongly agree, Slightly agree, Slightly disagree, Strongly disagree). We tested the validity of SCD against Kihon Checklist-Cognitive Function (KCL-CF) assessment (“Do your family or your friends point out your memory loss? E.g.‘You ask the same question over and over again.’”, “Do you make a call by looking up phone numbers?”, “Do you find yourself not knowing today’s date?”) and confirmed a significant association between SCD and KCL-CF (Pearson’s correlation coefficient > 0.4) [41] (Analyses using the outcome of KCL-CF was shown in supplementary materials). We categorized participants based on their responses into the cognitive decline group (Strongly agree and Slightly agree) and the no cognitive decline group (Slightly disagree, Strongly disagree).

#### ICT use

ICT use was assessed using two questions: ICT use history (start from the COVID-19 pandemic or before the pandemic or not using) and current ICT activities (sending e-mail; browsing; searching for information; communicate with others using LINE, Skype, ZOOM, etc.; using social networking service like Twitter, Facebook, Instagram, etc.). We deleted contradictory data within the two questions. Participants who reported either using the ICT since or before COVID-19 pandemic or chose at least one of the ICT activities were categorized into the ICT use group. Participants who reported “not using” and not chose any ICT activity for the second question were categorized into the non-ICT use group.

#### Loneliness and social isolation

Loneliness was assessed using the Japanese version of the Three-Item Loneliness Scale [42], which is a short version of Revised UCLA Loneliness Scale [43]. All items used a three-point scale (1: Hardly ever, 2: Some of the time, and 3: Often). Scores can range from 1 to 9, with higher scores representing higher loneliness levels. The scale has been shown to have high reliability and validity for Japanese, including older Japanese adults [42]. Cronbach’s α in this study was 0.70, indicating high internal consistency.

We adapted the approach of prior studies and then derived a social isolation index based on six items [44–46]. Participants were assigned one point if they (1) did not got married; (2) lived alone; (3) did not have a nearby supportive social network they can turn to when they need to talk to someone; (4) did not regularly meet with family or friends at normal times; (5) no weekly contact (less than one day a week) with family or friends by telephones or television telephone devices; (6) no weekly social participation (e.g. volunteering, activities with local organizations like a neighbourhood association, and participation in a local self-governing body). Scores ranged from 0 to 6, with higher scores indicating greater degrees of objective social isolation.

#### Other covariates

Sociodemographic covariates included gender (male or female), subjective economic status (1–3 scale indicating the higher point as a higher level), and education (1-5 scales indicating the higher point as a higher education). Physical activities were assessed by weekly sports activities including walking. Health-related covariates included smoking status (smoking, quitted or not smoking), comorbidities (none or at least one of the followings: dyslipidemia, heart disease, cerebrovascular disease, hypertension, and diabetes). Emotional state covariates consist depression (depressive tendency or not) and coronavirus anxiety (1–3 point). We applied depression-related items (5 items) from Kihon Checklist (KCL) to assess depression. KCL is a national-used screening scale of 25 items to evaluate comprehensive health outcomes including physical, psychological, and social domains [47]. Each response of difficulty with each item will be assigned one point. Using the suggested depressive outcome cut-off point of 2, we categorized the 2 of 5 depression-related items for depressive tendency group [48, 49]. Coronavirus anxiety score which is a sum of scores of perceived risks of getting infected with COVID-19 (1: high; 0: low) and death anxiety related to the infection (1: fear; 0: unfear).

### Statistical analysis

Descriptive analyses and hierarchical regression analyses were conducted for participants aged 70–79 years and ≥ 80 years separately. In each age group, differences in characteristics between the cognitive decline group and the no cognitive decline group were determined using the chi-square test for categorical variables and the t-test for continuous variables. Based on logistic regressions, Model 1 determined the main effects of poor social relationships (loneliness and social isolation) after controlled all the covariates. ICT use was added as moderator in Model 2. The interaction items between ICT use and the two social relationship variables were added in Model 3 and 4, respectively. We tested the improvement of model 2 to 4 by performing likelihood ratio tests [50, 51]. In addition, we conducted same analyses by using the score of KCL-CF as an objective assessment of cognitive function status to test the stability of our hypothesis (see supplementary materials) [41].

## Results

After excluding missing values for all the variables in the evaluation, a total of 706 older adults were included, which consisted of 484 participants aged 70–79 years and 222 participants aged ≥ 80 years. As shown in Table 1, SCD is more common in the ≥ 80 age group (21.6%) than in the 70–79 age group (12.0%). Participants who reported SCD felt more loneliness and with higher depression risk than participants with no cognitive decline in both age groups. Participants aged ≥ 80 years with SCD had less use of ICT and a higher social isolation score than participants with no cognitive decline.

**Table 1:** Characteristics of the study participants

	70–79 age group			≥ 80 age group		
	No Decline	Declined	Signif^1^	No Decline	Declined	Signif
N	426	58		174	48	
Age, mean ± SD	74.01 ± 2.79	74.84 ± 3.03	**	83.39 ± 2.84	84.21 ± 3.07	*
Gender (%)						
Male	53.3	50.0	n.s.	60.9	60.4	n.s.
Female	46.7	50.0		39.1	39.6	n.s.
Education, mean ± SD	3.77 ± 0.95	3.50 ± 0.86	**	3.60 ± 1.05	3.38 ± 1.27	n.s.
Economic Status, mean ± SD	2.04 ± 0.65	1.78 ± 0.68	***	1.98 ± 0.65	1.85 ± 0.62	n.s.
Social Isolation, mean ± SD	1.83 ± 0.95	1.97 ± 0.99	n.s.	1.94 ± 0.91	2.35 ± 1.19	***
Loneliness, mean ± SD	3.45 ± 0.97	4.17 ± 1.60	***	3.46 ± 1.03	4.15 ± 1.83	***
Coronavirus Anxiety, mean ± SD	1.36 ± 0.71	1.67 ± 0.57	***	1.36 ± 0.72	1.52 ± 0.62	n.s.
Depression (%)						
No	83.6	34.5	***	73.0	33.3	***
Yes	16.4	65.5		27.0	66.7	
Weekly sports (%)						
Yes	71.4	60.3	n.s.	58.0	45.8	n.s.
No	28.6	39.7		42.0	54.2	
Smoking (%)						
Smoking	4.2	6.9	n.s.	4.6	8.3	n.s.
Not Smoking	73.7	72.4		73.6	70.8	
Quited	22.1	20.7		21.8	20.8	
Has comorbidities^2^, (%)						
No	39.0	37.9	n.s.	28.7	29.2	n.s.
Yes	61.0	62.1		71.3	70.8	
ICT use (%)						
ICT-user	78.2	67.2	n.s.	54.0	31.3	**
Non-ICT user	21.8	32.8		46.0	68.8	

*SD* Standard deviation, *n.s.* No significance, *ICT* Information and communications technology

^1^Significantly different from the no decline group (^*^ *p* < 0.05 ^**^ *p* < 0.01 ^***^p < 0.001)

^2^comorbidities included none or at least one of the following: dyslipidemia, heart disease, cerebrovascular disease, hypertension, and diabetes

**Fig. 1:**
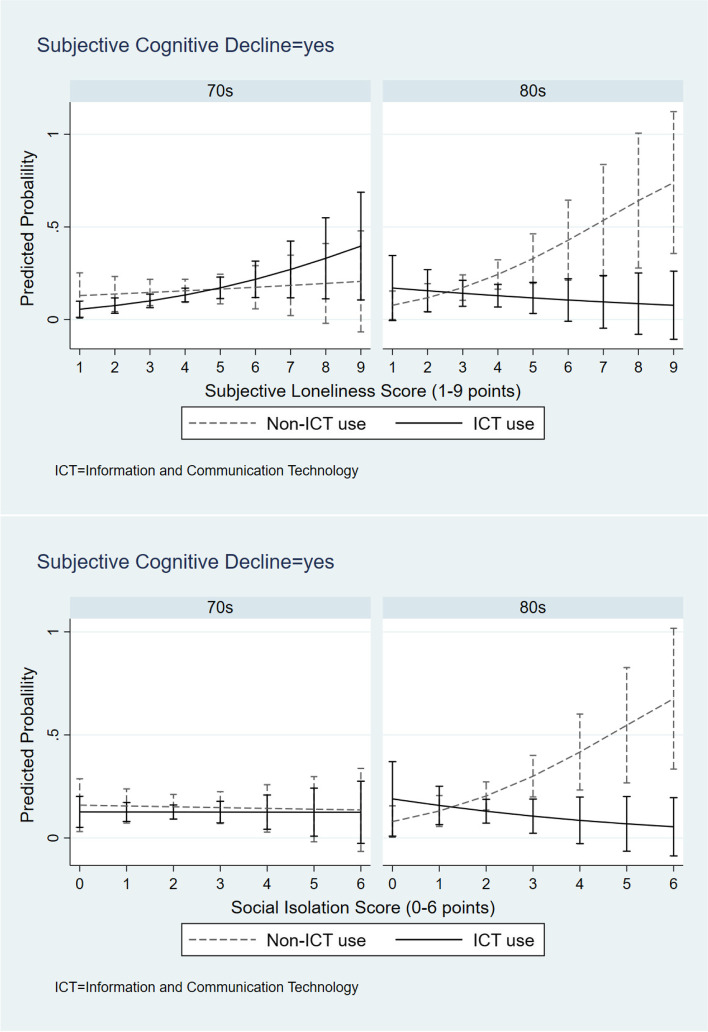
Interaction effects between ICT use and social relationships on cognitive decline by age group

As shown in Table 2, the results of Model 1 revealed that after controlling for covariates, loneliness (odds ratio [OR] = 1.30, 95% confidential interval [CI] = 1.01–1.69) and social isolation (OR = 1.44, 95% CI = 1.00–2.06) were significantly related to SCD for the participants aged ≥ 80 years. Model 2 showed a higher risk of SCD for non-ICT user than ICT user in the ≥ 80 age group (OR = 2.25, 95% CI = 1.01,5.01) and an improved model fit (Δ − 2 × log = -4.04, *p* = 0.04). Model 3 and 4 shown the interaction terms of ICT use by poor social relationships (loneliness, social isolation) significantly improved the model goodness fit (Δ − 2 × log = -5.12, *p* = 0.02 for Model 3; Δ − 2 × log = -4.7, *p* = 0.03 for Model 4) than Model 2 and both the interaction items significantly predicted SCD for the ≥ 80 age group (OR = 2.01, 95% CI = 1.05–3.85 for Model 3; OR = 2.45, 95% CI = 1.05–5.75 for Model 4). Only the association between loneliness and cognitive decline is shown in the 70–79 age group. For more details, the interaction effects are visualized in Fig. 1: Non-ICT users with high loneliness scores were more likely to experience SCD compared to ICT users with high loneliness. Similarly, older adults aged ≥ 80 years who were using ICT did not seem to be sensitive to the potential negative effect of social isolation on cognitive decline. Supplementary analyses revealed similar results that combinations of non-ICT use and poor social relationships were also related to objective cognition function performance (see Supplement Table 1 and Supplement Fig. 1).

**Table 2:** Results of the multilevel logistic regression analysis

	70–79 age group				≥ 80 age group			
	Model 1	Model 2	Model 3	Model 4	Model 1	Model 2	Model 3	Model 4
	Odds ratio [95% confidence intervals]				Odds ratio [95% confidence intervals]			
Loneliness Score	1.28*	1.28*	1.41*	1.28*	1.30*	1.32*	0.86	1.35*
	[1.01,1.63]	[1.00,1.62]	[1.05,1.89]	[1.00,1.62]	[1.01,1.69]	[1.01,1.73]	[0.52,1.45]	[1.03,1.78]
Social Isolation Score	0.98	0.98	0.98	0.98	1.44*	1.41 †	1.44 †	0.76
	[0.71,1.36]	[0.70,1.35]	[0.71,1.35]	[0.65,1.47]	[1.00,2.06]	[0.98,2.02]	[0.99,2.10]	[0.37,1.56]
Non ICT-user		1.21	3.79	1.23		2.25*	0.17	0.36
(ref: ICT-user)		[0.59,2.47]	[0.51,28.35]	[0.29,5.27]		[1.01,5.01]	[0.01,2.02]	[0.06,2.29]
Non ICT-user × Loneliness			0.75				2.01*	
(ref.: ICT-user × Loneliness)			[0.46,1.21]				[1.05,3.85]	
Non ICT-user × Social isolation				0.99				2.45*
(ref.: ICT-user × Social isolation)				[0.52,1.90]				[1.05,5.75]
N	484	484	484	484	222	222	222	222
− 2 LLR	278.18	277.92	276.52	277.92	194.24	190.2	185.08	185.5
△ − 2 LLR^a^		-0.26	-1.4	0		-4.04*	-5.12*	-4.7*

† *p* < 0.1, ^*^ *p* < 0.05. *ICT* Information and communications technology, *ref* Reference, *LLR* Likelihood ratio

Models were all adjusted for gender, education, economic status, coronavirus anxiety, depression, weekly physical activities, smoking status, and comorbidities. Model 2 = Model 1 + ICT use. Model 3 = Model 2 + ICT use × Loneliness. Model 4 = Model 3 + ICT use × Social Isolation

^a^results of likelihood ratio tests. Differences of -2LLR between Model 1&2, Model 2&3, Model 2&4

## Discussions

We examined the relationship between ICT use and SCD among Japanese older adults. We found that non-ICT users with high loneliness or social isolation scores were more likely to experience cognitive decline in the ≥ 80 age group after consideration of covariates, not in the 70–79 age group. It indicated a potential mitigating role of ICT use on the negative association between poor social relationship such as loneliness and social isolation, and cognitive decline in people aged ≥ 80 years.

The associations between ICT use and cognitive decline observed in this study corroborated previous studies [17, 18] and enriched data from Japan. Building a digital society has become one of the national strategies for dealing with social issues in Japan, an aging society [52]. A recent report showed that even though older adults are the fastest-growing group of ICT users, there is a digital divide between older adults and younger adults, with a lower rate of ICT use among older adults [52]. A study on the health-related outcomes of ICT use in older adults could contribute to reducing the digital divide by informing digital promotion-based public policies.

Even though the combined risks of non-ICT use and poor social relationships were first demonstrated in this study, we can find some possible explanations for the interaction effects from previous studies, as discussed in the introduction. However, the interaction effects of ICT use and loneliness or social isolation was age-sensitive. We only found the interaction effects in the ≥ 80 age group. We hypothesize that this may due to age differences in social resources. As we discussed, the influence of sociality on the cognitive reserve could be one potential pathway for the interaction effects of ICT and social relationships. However, there is a trend of decline in social resources by age [10]. In particular, the very old are more likely to live with a limited social network or less social support than the younger old [53]. Our results revealed that a higher loneliness or social isolation score was associated with a higher risk of cognitive decline for people aged ≥ 80 years, indicating that the very old group is more susceptible to the effect of poor social relationships [54]. As a result, limited social resources may affect strategies for coping with negative feelings for the very old people as well as the decline in capacity for self-regulation [31]. Traditional way for older adults to deal with loneliness feelings and network decline could be the acceptance [55, 56], but ICT use might work as a new tool. Thus, we conclude that the stimulation from ICT might be more meaningful to the very old population who were susceptible to limitations in social resources.

Emergencies like a pandemic could be a source of stress [57]. Beyond the life changes that come with shutdowns, older adults also need to cope with a higher risk of infection and death [58]. Studies from the Eastern context reported older adults’ emotional responses to the pandemic were more apparent considering the high mortality rate [59]. Thus, we controlled emotional health as covariates and conducted supplementary analyses using objective cognitive health outcome, which revealed the stability of our results. On the other hand, the decline of social reaction is common in daily life for older adults. Some studies also reported that older adults had fewer life changes than younger adults [60, 61]. Therefore, we suggested our findings is not only applied for an emergency context, but also applied for a normal context for older adults.

The findings in this study have practical implications. ICT has been used in medical practice as part of online treatment or training programs [34, 62]. We found that general informal ICT use among community dwellers in daily life could also have positive outcomes on cognitive function, especially in the very old population. Considering the effect of the digital divide on health and age-sensitive social vulnerability, information on ICT use among older adults in different contexts needs to be understood. However, we cannot conclude that ICT use is a must for cognitive intervention since several evidence from younger populations indicated that excessive ICT use has negative effects on cognitive health [63, 64]. More details on ICT use among older adults, like frequency or time, should be measured in the future. There is a possibility that those with cognitive decline did not respond to the questionnaire, which may affect our results. Another major limitation is that we could not infer causal directions between ICT use or the interaction effects and cognitive decline using our cross-sectional data. However, we cannot deny that ICT might affect cognitive decline since some studies found a bidirectional relationship between ICT use and cognitive function [16]. A longitudinal design is suggested to gather stronger evidence on causality. The high number of missing data is also a limitation of this study which mainly comes from the variable of ICT use items (15% missing); however, the missing cases of ICT use item were not found to be population with cognitive declined so it may not have affected overall results.

The major strengths of this study are the high response rate (71.6%) and a large random sample, which reduced nonresponse bias and suggests that our results are representative [65]. Data on ICT use among older adults is rare, especially data on the very old population. Our study is also highly relevant to the present situation and may contribute to with/post-pandemic era since poor social relationships are common during later life.

In conclusion, we have found a potential positive cognitive outcome of ICT use and a potential mitigating role of ICT use on the negative association between loneliness, social isolation, and cognitive decline in people aged ≥ 80 years. ICT use could be an effective solution to coping with poor social relationship-related cognitive decline for older adults with limited social resources. Further longitudinal design to study the intensity of ICT use and cognitive function is needed.

## Supplementary Information

**Additional file 1.**

## Data Availability

The datasets used and/or analyzed during the current study are available from the corresponding author on reasonable request.

## Abbreviations

ICT: Information and Communications Technology
SCD: Subjective Cognitive Decline
KCL: Kihon Checklist
KCL-CF: Kihon Checklist-Cognitive Function
