# Supplementary material for: Cognitive decline and poor social relationship in older adults during COVID-19 pandemic: can information and communications technology (ICT) use helps?
Source: BMC Geriatr. 2022 Apr 28;22:375. doi: 10.1186/s12877-022-03061-z (PMC9047378; doi:10.1186/s12877-022-03061-z)
Supplement: Supplementary file 1 — Additional file 1. [file 12877_2022_3061_MOESM1_ESM.docx]

Supplementary Table 1. Results of the multilevel liner regression analysis

|  | 70-79 age group | | | |  | ≥80 age group | | | |
| --- | --- | --- | --- | --- | --- | --- | --- | --- | --- |
|  | Model 1 | Model 2 | Model 3 | Model 4 |  | Model 1 | Model 2 | Model 3 | Model 4 |
|  | *B* | *B* | *B* | *B* |  | *B* | *B* | *B* | *B* |
| Loneliness Score (1-9) | 0.07** | 0.07** | 0.09** | 0.07** |  | 0.18*** | 0.18*** | 0.08 | 0.19*** |
|  |  |  |  |  |  |  |  |  |  |
| Social Isolation Score (1-6) | 0.05 | 0.04 | 0.04 | 0.04 |  | 0.12* | 0.11* | 0.11* | -0.13 |
|  |  |  |  |  |  |  |  |  |  |
| Non ICT-user (ref: ICT-user) |  | 0.06 | 0.29 | 0.04 |  |  | 0.38*** | -0.30 | -0.40 |
|  |  |  |  |  |  |  |  |  |  |
| Non ICT-user × Loneliness |  |  | -0.06 |  |  |  |  | 0.19** |  |
| (ref.: ICT-user × Loneliness) |  |  |  |  |  |  |  |  |  |
|  |  |  |  |  |  |  |  |  |  |
| Non ICT-user × Social isolation |  |  |  | 0.01 |  |  |  |  | 0.39*** |
| (ref.: ICT-user × Social isolation) |  |  |  |  |  |  |  |  |  |
|  |  |  |  |  |  |  |  |  |  |
| N | 535 | 535 | 535 | 535 |  | 259 | 259 | 259 | 259 |
| Adjusted R^2^ | 0.109 | 0.111 | 0.113 | 0.111 |  | 0.208 | 0.247 | 0.267 | 0.292 |

Note. *B* =Unstandardized coefficients; * p<0.05, ** p<0.01, *** p<0.001. ICT= information and communications technology. ref. = reference. LLR= likelihood ratio. Adjusted R^2^= Adjusted R-squared. Models were all adjusted for gender, education, economic status, coronavirus anxiety, depression, smoking status, weekly physical activities, and comorbidities. Model 2 = Model 1+ICT use. Model 3 = Model 2+ ICT use × Loneliness. Model 4 = Model 3 + ICT use × Social Isolation.

Supplementary Figure 1. Interaction effects between ICT use and social relationship on cognitive status by age groups.


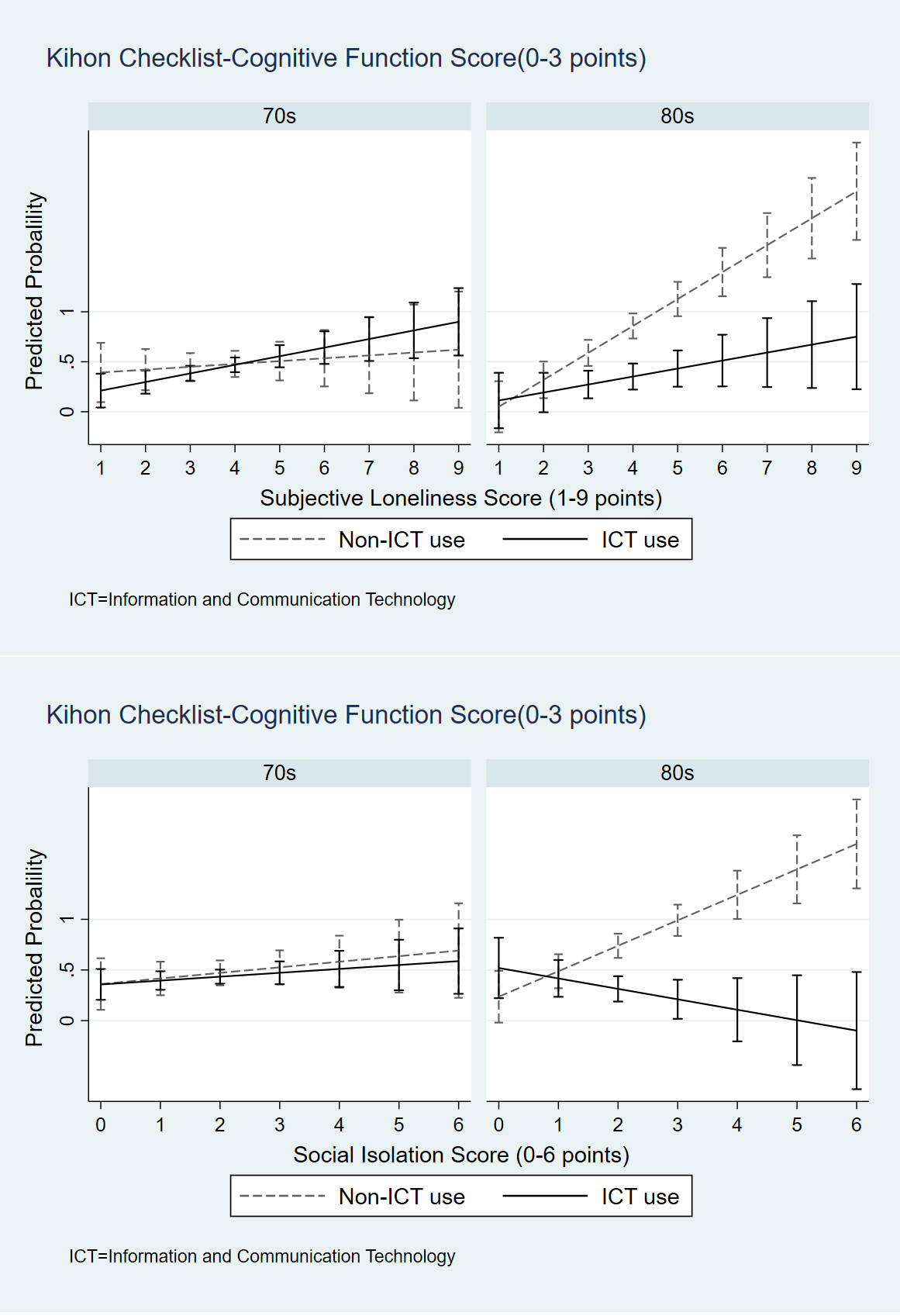


Note. ICT= Information and Communication Technology.
